# Phenotypic differences of atopic dermatitis stratified by age

**DOI:** 10.1016/j.jdin.2022.08.026

**Published:** 2022-10-10

**Authors:** Sheena Chatrath, Jonathan I. Silverberg

**Affiliations:** a Department of Dermatology, The George Washington University School of Medicine and Health Sciences, Washington, District of Columbia; b Department of Dermatology, Feinberg School of Medicine at Northwestern University, Chicago, Illinois

**Keywords:** Atopic dermatitis, eczema, patient-reported outcomes, physical activity, severity, AD, atopic dermatitis

## Abstract

**Background:**

Atopic dermatitis (AD) is common across all ages. Understanding heterogeneous age-related phenotypes may improve AD management.

**Objective:**

To determine age-related clinical phenotypes of AD.

**Methods:**

A prospective, dermatology practice-based study was performed (*n* = 380). AD severity was evaluated using questionnaires and full-body examination. Phenotypes were determined using latent class analysis.

**Results:**

There were 23 (6.1%) pediatric patients (<18 years), 176 (46.3%) young adults (18-39 years), and 181 (47.6%) older adults (≥ 40 years). Both young and older adults experienced less AD on ankles (adjusted odds ratio [95% confidence interval]: 0.41 [0.19-0.90], 0.43 [0.20-0.94]), moderate-severe AD on flexures (0.47 [0.26-0.87], 0.30 [0.16-0.56]), pityriasis alba (0.24 [0.11-0.52], 0.07 [0.03-0.18]), oozing lesions (0.44 [0.25-0.79], 0.35 [0.20-0.63]), moderate-severe excoriations (0.49 [0.28-0.85], 0.44 [0.26-0.76]), and severe itch (adjusted β [95% confidence interval], −1.46 [−2.63 to −0.29]; −1.79 [−2.94 to −0.65]) compared with pediatric patients. Young adults experienced more AD around the eyes (2.92 [1.21-7.02]). Older adults experienced more AD on elbows (0.34 [0.19-0.64]), nipples (0.40 [0.16-0.99]), knees (0.27 [0.14-0.53]), keratosis pilaris (0.38 [0.15-0.98]), and lichenification (0.47 [0.22-0.98]). Four classes were identified for distribution of AD and associated signs.

**Conclusion:**

Distinct phenotypes exist by age with younger patients experiencing more AD signs and symptoms. Clinicians should consider them when managing AD.

**Capsule Summary**

• Distinct phenotypes exist by age with older adults presenting with less flexural eczema and fewer associated signs of atopic dermatitis.
• Clinicians should recognize the various clinical phenotypes of atopic dermatitis and consider a personalized approach for the management of atopic dermatitis.

## Introduction

Atopic dermatitis (AD) is a chronic inflammatory skin disorder affecting ∼20% of children, 10% adults, and 1-3% of geriatric patients.^1^^,^^2^ Recent studies suggest the clinical characteristics of AD differ across age groups.^3^, ^4^, ^5^, ^6^ In a systemic review and meta-analysis, patients from pediatric studies were found to have AD involvement of the eyelid, auricular area, and wrist, whereas patients from adult studies were found to have hand and foot involvement as well as chronic signs of AD.^3^

The heterogeneity of AD^7^ has led to the identification of various clinical phenotypes and a movement toward personalized medicine in dermatology. Previous studies have found differences in the clinical characteristics of AD depending on age of AD onset, ethnic background, and AD severity.^3^^,^^8^^,^^9^ However, none have prospectively compared the clinical characteristics and associated signs by age group. Improved understanding of the clinical phenotypes of AD may help guide choice of treatment and improve health outcomes. In this study, we sought to identify the phenotypic differences between pediatric, young adult, and older adult patients with AD.

## Methods

### Study design

The study was approved by the institutional review board at Northwestern University. A prospective dermatology practice-based study was performed between 2013 and 2019 that included patients with a diagnosis of AD as defined by the Hanifin-Rajka diagnostic criteria.^10^ Participants were recruited from an eczema clinic at an academic medical center. Exclusion criteria included those with an alternative diagnosis (determined by epicutaneous patch testing and skin biopsy), lack of definitive diagnosis (determined by the Hanifin-Rajka criteria), or the inability to complete questionnaires. Patients were categorized into 1 of 3 age groups: “Pediatric” (0-17 years), “Young adult” (18-39 years), and “Older adult” (≥ 40 years). Participation was formally assessed where virtually all (>99%) patients who were invited agreed to participate.

### Outcome measures

Data were collected using self-administered electronic questionnaires before the appointment. Questionnaires included age of AD onset, sociodemographics, Visual Analog Scale itch and sleep for Scoring AD (1 question each, range 0-10^11^), and Numeric Rating Scale for skin pain and itch (range 0-10).^12^^,^^13^ AD severity was assessed using Eczema Area Severity Index (0 = clear, 0.1-5.9 = mild, 6.0-22.9 = moderate, 23.0-72 = severe)^14^. A full body skin exam was performed by a dermatologist (J.S.). The distribution of AD involvement (scalp, eyelids, neck, nipples, elbows, wrists, hands, fingers, knees, genitalia, ankles, and feet) as well as associated signs (follicular eczema, nummular eczema, ichthyosis, keratosis pilaris, oozing lesions, palmar hyperlinearity, and xerosis) were assessed.

### Statistical analysis

All statistical analyses were performed using SAS 9.4 (SAS Institute). Summary statistics for baseline characteristics were performed. Repeated-measure, binary logistic regression models were constructed to analyze the relationship between age and the various clinical manifestations of AD. The dependent variables were the geographical distribution of AD lesions (scalp, face, neck, chest, upper, and lower extremities), AD signs (lichenification, skin oozing, swelling, excoriation, and redness), and associated signs (xerosis, keratosis pilaris, ichthyosis, palmar hyperlinearity, nummular, and follicular eczema). Repeated-measure, linear regression models were constructed to analyze the relationship between age and AD symptoms. The dependent variables were Numeric Rating Scale average-itch or skin pain. Multivariable models were adjusted for race and sex. Adjusted odds ratios, beta, and 95% confidence intervals (CIs) were estimated. A *P* value of <.05 was considered statistically significant.

Latent class analysis was used to examine the phenotypic patterns of 8 signs and 8 sites of AD lesions. The conditional probabilities were estimated using maximum likelihood to characterize the latent classes. Conditional probabilities indicate the chance that a patient would have that feature, with probabilities closer to 0 indicating lower chances, and probabilities closer to 1 indicating higher chances. The number of latent classes was selected using Akaike’s Bayesian Information Criterian.

## Results

### Baseline characteristics

Overall, 380 patients were recruited in the study, including 234 (61.6%) females, 176 (46.3%) adults between 18 and 39, and 181 (47.6%) adults 40 years or older (Table I). The mean ± standard deviation age of AD onset was 18.4 ± 28.9. At baseline, patients commonly reported history of asthma (41.9%), history of hay fever (48.9%), and other food allergies (35.4%); most had mild (47.0%) or moderate (33.8%), followed by severe (11.6%) or clear/almost clear (7.5%) AD determined by EASI score.

**Table I:** Baseline characteristics of the cohort

Baseline characteristics	Value
Age (y) - Freq (%)	
<18	23 (6.05%)
18-39	176 (46.32%)
40+	181 (47.63%)
Sex - Freq (%)	
Male	146 (38.42%)
Female	234 (61.58%)
Age of AD onset - Mean ± SD	18.4 ± 28.89
Race - Freq (%)	
Non-White	141 (37.11%)
White	239 (62.9%)
History of asthma - Freq (%)	
No	214 (58.15%)
Yes	154 (41.85%)
History of hay fever - Freq (%)	
No	166 (51.08%)
Yes	159 (48.92%)
History of food allergies - Freq (%)	
No	208 (64.60%)
Yes	114 (35.40%)
Baseline EASI - Mean ± SD	9.6 ± 11.7
Baseline Numeric Rating Scale itch - Mean ± SD	5.3 ± 3.2

*AD*, Atopic dermatitis.

**Table II:** Summary of associations of AD lesional distribution, morphology, and symptoms with age

Distribution of AD lesions	Age, y	Effect	Morphology of lesions and associated signs of AD	Age	Effect	AD symptoms	Age, y	Effect
Scalp	18-39		Follicular eczema	18-39		Skin pain	18-39	
	40+			40+			40+	−
Around the eyes	18-39		Ichthyosis	18-39	−	Itch	18-39	
	40+	−		40+	−		40+	
Postauricular	18-39		Keratosis Pilaris	18-39	−			
	40+			40+				
Face	18-39		Nummular eczema	18-39				
	40+			40+				
Neck	18-39		Palmar hyperlinearity	18-39				
	40+			40+				
Nipples	18-39		Pityriasis Alba	18-39				
	40+			40+				
Elbows	18-39		Lichenification	18-39				
	40+			40+				
Flexural sites	18-39		Ooze	18-39				
	40+			40+				
Wrists	18-39		Redness	18-39				
	40+			40+				
Hands	18-39		Excoriation	18-39				
	40+			40+				
Fingers	18-39		Swelling	18-39				
	40+			40+				
Genitalia	18-39	−	Xerosis	18-39				
	40+	−		40+	−			
Knees	18-39		Cheilitis	18-39				
	40+			40+				
Ankles	18-39							
	40+							
Feet	18-39							
	40+							

*AD*, Atopic dermatitis.

indicates an adjusted odds ratio of ≥4.000 or adjusted beta coefficient of ≥2.000;  indicates an adjusted odds ratio of 2.000 to 3.999 or adjusted beta coefficient of 0.5000 to 1.999.

– indicates an adjusted odds ratio of 1.000 to 1.999 or adjusted beta coefficient of -0.4999 to 0.4999.

indicates an adjusted odds ratio of 0.751 to 0.999 or adjusted beta coefficient of -0.5000 to -1.999;  indicates an adjusted odds ratio of ≤0.75 or adjusted beta coefficient of ≤ -2.000.

### Distribution of AD lesions

**Fig 1:**
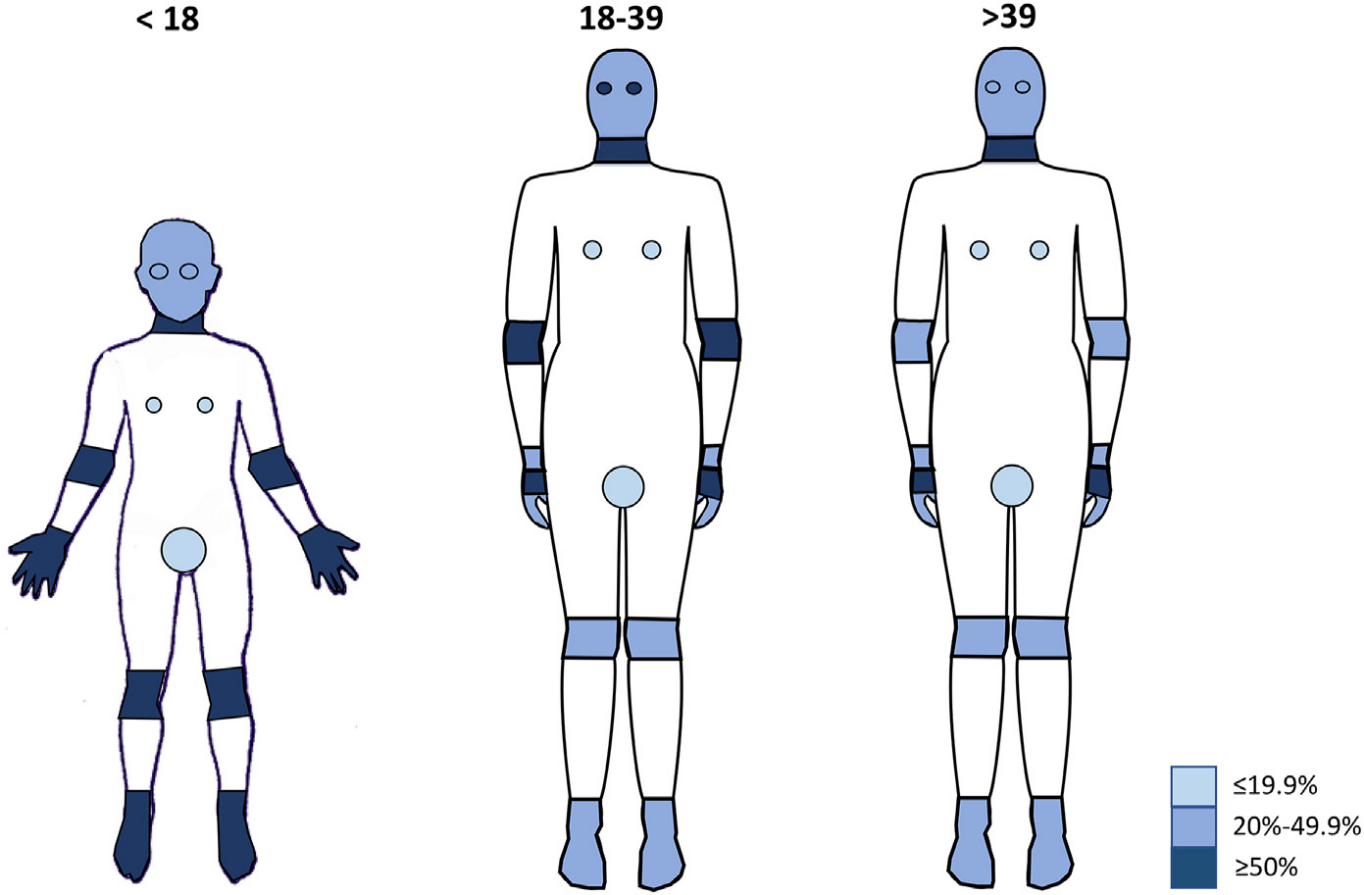
Distribution of AD lesions in patients <18 years old, 18-39 years old, and >39 years old. The scale represents the percent of patients affected in the area. *AD*, Atopic dermatitis.

The most common sites of AD were hands (75.0%), fingers (67.9%), and flexures (63.3%) in pediatric patients (0-17 years); flexures (57.5%), anterior neck (56.8%), and elbows (54.3%) in young adults (18-39 years); anterior neck (59.7%), hands (57.9%), and around the eyes (48.5%) in older adults (≥ 40 years) (Fig 1).

Compared with pediatric patients, young adults were less likely to report AD lesions on the ankles (adjusted odds ratio [95%CI]: 0.41 [0.19-0.90]), but more likely to develop AD around the eyes (2.92 [1.21-7.02]). Older adults were less likely to report AD on the nipples (0.40 [0.16-0.99]), elbows (0.34 [0.19-0.64]), flexures (0.48 [0.28-0.83]), knees (0.27[0.14-0.53]), and ankles (0.43 [0.20-0.94]). In addition, in comparison to pediatric patients, young adults were less likely to have moderate-severe AD lesions on the flexures (0.47 [0.26-0.87]). Older adults were less likely to have moderate-severe AD on flexures (0.30 [0.16-0.56]), as well as the postauricular area (0.47 [0.20-0.98]), (Table II, Supplementary Tables 1-3, available via Mendeley at https://data.mendeley.com/datasets/5z56x8fvkg).

### AD signs

The most common signs of AD among pediatric, young, and older adults were redness (94.1%, 94.3%, 90.0%), lichenification (92.6%, 84.8%, 78.5%), and excoriations (85.2%, 80.6%, 77.9%) respectively. Compared with pediatric patients, young adults were less likely to have pityriasis alba (0.24 [0.11-0.52]) and oozing lesions (0.44 [0.25-0.79]) on physical exam, but no differences in cheilitis, follicular eczema, ichthyosis, keratosis pilaris, nummular eczema, palmar hyperlinearity, lichenification, redness, excoriations, swelling, or xerosis. Older adults were also less likely to have pityriasis alba (0.07 [0.03-0.18]) and skin oozing (0.35 [0.20-0.63]) as well as lichenification (0.47 [0.22-0.98]), and keratosis pilaris (0.38 [0.15-0.98]).

Younger adults were less likely to have moderate-severe excoriations (0.49 [0.28-0.85]), whereas older adults were less likely to have moderate-severe pityriasis alba (0.05 [0.00-0.54]), lichenification (0.50 [0.28-0.88]), skin oozing (0.27 [0.27-0.88]), and excoriations (0.44 [0.26-0.76]). (Table II, Supplementary Tables 3-5, available via Mendeley at https://data.mendeley.com/datasets/5z56x8fvkg).

### AD symptoms

Severe itch was most commonly reported in children (47.1%), followed by young adults (43.4%), and older adults (38.6%). Compared with pediatric patients, both younger adults (aβ [95%CI]: −1.46 [−2.63 to −0.29]) and older adults (−1.79 [−2.94 to −0.65]) had less severe itch (Table II, Supplementary Table 3, available via Mendeley at https://data.mendeley.com/datasets/5z56x8fvkg).

Skin pain was most common in younger adults (41.7%) followed by older adults (38.1%), and least common in children (28.6%). Severe levels of pain were most common in older adults (18.0%), whereas moderate levels were most common in young adults and children (15.2%, 14.3%). Severity of skin pain was similar between children and young adults (0.51 [−0.57 to 1.60]) or older adults (0.23 [−0.85 to 1.20]) (Table II, Supplementary Table 3, available via Mendeley at https://data.mendeley.com/datasets/5z56x8fvkg).

### Latent class analysis

**Fig 2:**
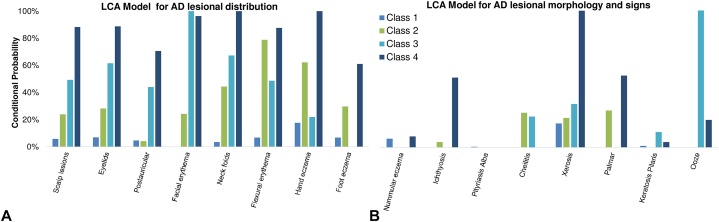
Latent class analysis. **A**, Latent class analysis identified 4 distinct classes for the distribution of AD lesions from 8 body sites. The probability of each body area belonging to the distinct classes is shown. Class 1 shows low probabilities of all AD sites; class 2 shows low probability of face and feet involvement, and intermediate probabilities for other AD sites; class 3 shows high probability of face involvement, low probability of hand and feet involvement, and intermediate probability of all other AD sites; and class 4 shows intermediate probability of postauricular and feet involvement, and high probabilities of all other AD sites examined. **B**, Four distinct classes were also identified from 8 signs and symptoms of AD. Class 1 shows zero-low probabilities of all AD signs; class 2 shows low probabilities of all AD signs; class 3 shows high probability of oozing lesions and low probabilities of all other signs; and class 4 shows high probabilities xerosis, intermediate probabilities of ichthyosis and palmar hyperlinearity, and low probabilities of all other signs. *AD*, Atopic dermatitis.

Latent class analysis was used to identify the phenotypic patterns of AD lesional distribution and associated signs (*n* = 380). The best-fit model was determined by minimal Akaike’s Bayesian Information Criterian and interpretability. Conditional probabilities of the various sites and symptoms in each class are plotted in Fig 2.

### Distribution of AD lesions

Four classes were identified for distribution of AD lesions. Class 1 had low probabilities of AD involvement at all sites examined. Class 2 had low probabilities of scalp, face, and foot involvement, and intermediate probability of all other AD sites. Class 3 had low probabilities of hand and foot involvement, high probability of facial erythema, and intermediate probability of all other AD signs. Class 4 had intermediate probability of postauricular and foot involvement, and high probability of all other AD sites examined. Pediatric patients were most commonly in class 4 (33.3%), followed by classes 1 and 2 (26.7%), and least commonly in class 3 (13.3%). In young adults, classes 4 and 1 were most common (32.4%, 29.4%), followed by class 2 (27.9%), and least commonly class 3 (10.3%). In older adults, class 1 was most common (40.3%), followed by class 4 (23.6%), and least commonly classes 2 and 3 (18.1%).

### AD signs

Four latent classes were identified for the signs and symptoms of AD. Class 1 had zero-low probability of all AD signs; class 2 had low probability of all AD signs; class 3 had high probability of oozing lesions, and low probability of all other signs; and class 4 had high probability of xerosis, intermediate probability of ichthyosis and palmar hyperlinearity, and low probability of all other AD signs. The most common class across all age groups was class 1, affecting 85.6% of older adults, 81.8% of younger adults, and 82.6% of pediatric patients. In pediatric patients, class 3 was the second most common (8.7%), followed by classes 2 and 4 (4.4%). In young adults, 9.7% were in class 2, 5.7% class 4, and 2.8% were in class 3. In older adults, 8.3% were in class 4, 4.4% in class 2, and 1.67% in class 3.

### Discussion

This study demonstrated that the phenotypic presentation of AD varies across age groups. Consistent with previous studies, pediatric and adult patients had similar likelihood of xerosis, head and neck involvement, cheilitis, and nummular eczema.^3^^,^^15^ Additionally, we found similar likelihood of AD involving the fingers, wrist, hands, genitalia, feet as well as the associated signs: follicular eczema, ichthyosis, excoriations, swelling, and redness. However, we found increased likelihood of AD lesions around the eye in young adults as well as decreased likelihood of moderate-severe lichenification in older adults. This is in contrast to a previous study that found increased prevalence of eye involvement in pediatric patients and increased lichenification in adult patients with AD.^3^ These results may be because of differences in the prevalence of respiratory atopy and environmental exposures.

In older adults, AD was previously shown to present similarly as adolescents and young adults with respect to presence of AD lesions on elbow and knee folds, face and neck, and presence of Dennie-Morgan lines, and nummular eczema lesions.^16^ We found that older adults age ≥ 40 years, but not young adults, were less likely to develop AD on the nipples and flexures. They also were less likely to have the associated signs keratosis pilaris and lichenification, as well as less severe signs than younger patients. Our results suggest that clinical manifestation of AD varies with age in previously unrecognized ways; clinicians should account for these differences when evaluating patients of different age groups.

Itch is the most common and burdensome symptom of AD.^17^ Consistent with previous studies, we found that itch is ubiquitous across all age groups. However, pediatric patients were more likely to experience severe itch compared to younger and older adults. More severe itch was likely related to more extensive AD lesions compared to adult patients. Interestingly, despite increased itch in pediatric patients, we found no difference in the severity of skin pain across all age groups. Moreover, pediatric patients reported skin pain less often than adult patients. This may be because of age-related differences of pain perception. For example, one study found that children tend to perceive severe itch and pain as the same sensation, whereas adults perceive these symptoms separately.^18^

Previous studies found differences of AD presentation by age of AD onset, AD severity, ethnicity, and progression throughout childhood.^6^^,^^8^^,^^9^ Our study identified an additional subtype of AD, with distinct differences across age. Consistent with the Hanifin-Rajka major criteria, all age groups experienced significant pruritus, however, both young and older adults were less likely than pediatric patients to experience several minor criteria. This wide variation in presentation may make the diagnosis of AD difficult.^19^ In pediatric patients, acute contact dermatitis, scabies, molluscum dermatitis, tinea corporis, psoriasis, and actinic prurigo may mimic AD and should be ruled out. In adult patients, psoriasis, cutaneous T-cell lymphoma, drug eruptions, contact dermatitis, scabies, tinea corporis, dermatomyositis, and polymorphous light eruption should be considered as part of the differential diagnosis.^19^^,^^20^ The broad differential diagnosis is particularly important in adult patients without flexural eczema or with hand or facial dermatitis.

Despite the growing evidence of unique AD phenotypes, treatment is currently based on AD severity.^19^ Although recent treatments such as dupilumab have been a turning point for many patients, there are still a subset of patients who are partial or complete nonresponders.^21^^,^^22^ Although it is unclear why some patients do not respond, it may be due in part to their subtype of AD. Though, 1 open-label Italian observational study of 221 patients treated with dupilumab found that it was effective across multiple AD phenotypes, including nummular eczema, prurigo, and erythroderma.^23^ Further studies are needed to identify whether more phenotypes of AD exist, how these phenotypes relate to biomarkers of disease, and what treatment regimen is best suited for each of the subtypes.

This study has several strengths, including the large sample size, standardized assessment of AD signs, and prospective design. Previously validated patient-reported measures were used for AD severity.^24^ Latent class analysis was used to identify the dominant phenotypes of AD across age groups, with 4 subgroups identified for both AD sites and signs. The limitations of this study include recruitment of patients from a single academic center that may limit generalizability, lack of data on treatment effects on the various phenotypes, and lower proportion of pediatric patients in the cohort. Future even larger multicenter studies are warranted.

In conclusion, the clinical presentation of AD differs between children, young adults, and older adults. In particular, pediatric patients are more likely to experience AD in classical sites such as the knees and have more associated and severe signs of disease. Older adults tend to present with less flexural eczema and the fewest associated signs, potentially making the diagnosis difficult in this population. Clinicians should recognize the various clinical phenotypes of AD and consider a personalized approach for the management of AD. Further research is needed to understand how these distinct phenotypes respond to current treatment options.

## Conflicts of interest

None disclosed.
